# Human genetics of meningococcal infections

**DOI:** 10.1007/s00439-020-02128-4

**Published:** 2020-02-17

**Authors:** Stephanie Hodeib, Jethro A. Herberg, Michael Levin, Vanessa Sancho-Shimizu

**Affiliations:** 1 Department of Paediatric Infectious Disease, Faculty of Medicine, Imperial College London, Norfolk Place, London, W2 1PG UK; 2 Department of Virology, Faculty of Medicine, Imperial College London, Norfolk Place, London, W2 1PG UK

## Abstract

*Neisseria meningitidis* is a leading cause of bacterial septicaemia and meningitis worldwide. Meningococcal disease is rare but can be life threatening with a tendency to affect children. Many studies have investigated the role of human genetics in predisposition to *N. meningitidis* infection. These have identified both rare single-gene mutations as well as more common polymorphisms associated with meningococcal disease susceptibility and severity. These findings provide clues to the pathogenesis of *N. meningitidis,* the basis of host susceptibility to infection and to the aetiology of severe disease. From the multiple discoveries of monogenic complement deficiencies to the associations of complement factor H and complement factor H-related three polymorphisms to meningococcal disease, the complement pathway is highlighted as being central to the genetic control of meningococcal disease. This review aims to summarise the current understanding of the host genetic basis of meningococcal disease with respect to the different stages of meningococcal infection.

## Introduction

*Neisseria meningitidis *(*Nm*) is a common commensal bacterium that is paradoxically also a devastating human pathogen. It is a Gram-negative diplococcus that selectively colonises the human nasopharynx (Stephens et al. 1983). *Nm* is encapsulated with a polysaccharide capsule which can be classified into 13 capsular serogroups known to cause disease. The six major serogroups typically associated with disease are serogroups A, B, C, Y, W, and X (Rosenstein et al. 2001; Xie et al. 2013). Carriage of *Nm* refers to the commensal colonisation of the bacterium in the human nasopharynx, whereas invasive meningococcal disease (IMD) is a result of bacterial invasion of the mucosal layer leading to its dissemination throughout the body causing meningitis and/or septicaemia, and may result in purpura fulminans and/or death (Coureuil et al. 2012; Lecuyer et al. 2017; Pace and Pollard 2012). Carriage rates vary depending on multiple variables including age, geographical location, and living conditions but is estimated at 10% in the general population (Cartwright et al. 1987; Caugant et al. 1992). Whilst majority of carriers remain asymptomatic or can develop low-grade bacteraemia, carriage of *Nm* leads to the production of protective antibodies and development of acquired immunity, and very rarely leads to invasive disease (Caugant and Maiden 2009; Goldschneider et al. 1969a; b; Pace and Pollard 2012). Incidence of IMD resulting in meningitis and septicaemia is estimated at 1–3 cases per 100,000 individuals in Europe and North America (Parikh et al. 2018; Yazdankhah and Caugant 2004). However, in the “meningitis belt” of sub-Saharan Africa, attack rates during epidemics can reach 1000 cases per 100,000 individuals (Yazdankhah and Caugant 2004). The reasons for these regional differences in IMD rates are not fully understood; however, non-genetic environmental factors have been suggested to play a role (Agier et al. 2013; Omoleke et al. 2018). Young children are at particular risk of developing IMD due to the absence of protective antibodies. Whilst disease rates are high in those under 5 years of age, there are other peaks of IMD incidence seen in adolescents and in old age (Caugant and Maiden 2009; Cohn et al. 2013; Rosenstein et al. 2001). IMD is rare but it causes significant mortality at an overall rate of 10–15% with up to 19% of survivors suffering from severe life-long sequalae with a reduced quality of life (Cohn et al. 2013; Erickson and De Wals 1998; Kirsch et al. 1996; Pace and Pollard 2012).

Human genetics is known to influence response to pathogens. Nucleotide variants that alter or abolish the function of immune-related genes are important determinants of susceptibility to infection and course of disease (Casanova 2015a, b). Human genetic investigations are particularly pertinent to *Nm* infections as *Nm* is a human-host restricted pathogen resulting in a lack of suitable animal models. Due to this host restriction, it is anticipated that all evolutionary adaptations of the pathogen over time must be specific to human responses (Laver et al. 2015). Multiple genes have been identified via familial linkage, genome-wide association studies (GWASs), and candidate gene-based studies to influence the course of infection, elucidating the key pathways involved in IMD and the impact of the role of genetics (Brouwer et al. 2010; Casanova 2015b; Wright et al. 2009). A study of sibling risk ratio for IMD, comparing the risk of disease within family members to the general population, showed that host genetics contributed to approximately 30% of the total risk of developing disease (Haralambous et al. 2003). Monogenic disorders of the complement pathway have long been known to predispose to IMD (Westberg et al. 1995). Furthermore, GWASs for infection susceptibility are well established as a method for identification of more common polymorphisms for instance, polymorphisms of complement factor H (*CFH*) and complement factor H-related 3 (*CFHR3*) have been associated with IMD, highlighting the host genetic contribution to disease (Davila et al. 2010; Martinon-Torres et al. 2016).

**Fig. 1:**
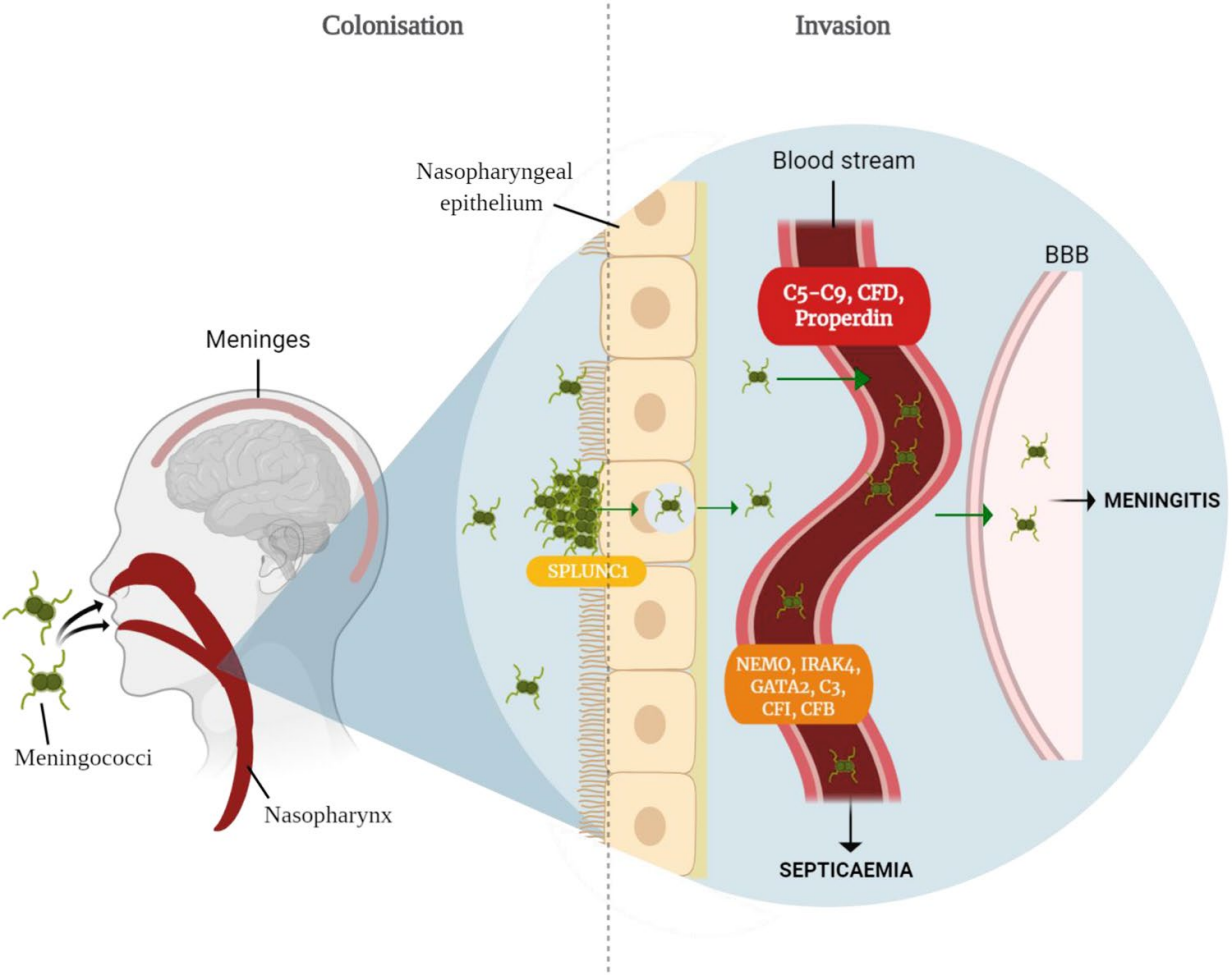
Monogenic disorders underlying *Neisseria meningitidis* infection. *Nm* is transmitted via droplets and selectively colonises the human nasopharynx. In susceptible hosts, meningococci can invade and cross the nasopharyngeal mucosal epithelium to gain access to the blood stream. Once inside the bloodstream the meningococci grow in number and are disseminated throughout the host. Uncontrolled growth in the blood, leads to high titres of *Nm* and septicaemia. In other patients, there is less *Nm* replication in the blood, but meningococci breach the blood brain barrier (BBB), multiply uncontrollably in the cerebrospinal fluid and infect the meninges, leading to meningitis. The genes highlighted in red are monogenic disorders almost exclusively associated with IMD. Highlighted in orange, are monogenic disorders associated with bacterial infections and, though not exclusive to *Nm* infection, has been observed in cases of IMD. *SPLUNC1*, highlighted in yellow, has recently been demonstrated as a monogenic disorder associated with IMD though its pathogen exclusivity is unknown. Image created with biorender.com

This review describes the role of human genetics with respect to the different stages of *Nm* infection. This includes the initial meningococcal colonisation of the human nasopharynx, followed by penetration of the mucosal membrane and invasion of the bloodstream, ultimately leading to systemic complications that can arise from an abnormal inflammatory and coagulation response. We have considered aspects of the immune system that are functionally related and grouped together in themed sections, whilst we acknowledge that these categorizations are not definitive and some genes may be involved in various stages of meningococcal pathogenesis. This review will summarise the contribution of host genetics at each phase of meningococcal infection highlighting the genes either associated with IMD or responsible for the monogenic disorders that determine IMD (Fig. 1).

## Colonisation

*Nm* selectively colonises the epithelial surface of the nasopharynx. Initial adhesion is mediated by the meningococcal type IV pili and then further facilitated by interaction of its Opacity-associated adhesion (Opa) proteins with host cell surface proteins including carcino-embryonic antigen cell adhesion molecule (CEACAM) proteins found on the nasopharyngeal epithelium (Carbonnelle et al. 2006; Virji 2009; Virji et al. 1996). Host colonisation is commonly asymptomatic; however, in some cases, colonisation can lead to invasion of the protective mucosa and entry of meningococci into the bloodstream, resulting in IMD (Aycock and Mueller 1950; Virji 2009). A candidate gene-based study found specific haplotypes in *CEACAM3* and *CEACAM6* associated with IMD indicating that CEACAM proteins are key factors in initial meningococcal infection (Table 2) (Callaghan et al. 2008). A recent study reported a novel heterozygous mutation in short palate, lung, and nasal epithelial clone 1 (*SPLUNC1,* also known as *BPIFA1*) in three IMD cases (Table 1). This autosomal dominant *SPLUNC1* mutation affected meningococcal biofilm formation, colonisation, and subsequent invasion, and is the first monogenic gene study demonstrating control of *Nm* colonisation (Mashbat et al. 2019).

**Table 1:** Monogenic disorders associated with meningococcal disease

Gene	Variant	Inheritance	Study type	Gene-specific phenotype	Infection phenotype	Disease outcome	References
**COLONISATION**							
* SPLUNC1*	c.65G > A, p.G22E	AD	Familial	Increased bacterial adhesion	IMD	Susceptibility	Mashbat et al. (2019)
**INVASION**							
* CFP*	c.481C > T, p.R161X	XR	Familial	Reduced complement function	IMD	Susceptibility	Westberg et al. (1995)
	c.1240T > G, p.Y414D	XR	Familial	Reduced complement function	IMD	Susceptibility	Fredrikson et al. (1996)
	c.617C > G, p.S206X	XR	Familial	Reduced complement function	IMD	Susceptibility	van den Bogaard et al. (2000)
	c.893G > T p.G298V	XR	Familial	Reduced complement function	IMD	Susceptibility	van den Bogaard et al. (2000)
	c.1164G > A, p.W388X	XR	Familial	Reduced complement function	IMD	Susceptibility	Helminen et al. (2012)
* C5*	c.1055A > G, p.Y352C	AR	Familial	Reduced complement function	IMD	Susceptibility	Marujo et al. (2019)
	c.754G > A, p.A252T	AR	Familial	Reduced complement function	IMD	Susceptibility	Owen et al. (2015)
	c.55C > T, p.Q19X; c.4444C > T, p.R1482X^a^	AR	Familial	Reduced complement function	IMD	Susceptibility	Wang et al. (1995)
	c.1115A > G, p.K372R	AR	Familial	Reduced complement function	IMD	Susceptibility	Pfarr et al. (2005)
	c.4890-4891delinsG, p.L1631fs	AR	Familial	Reduced complement function	IMD	Susceptibility	Delgado-Cervino et al. (2005)
* C6*	c.878delA	AR	Familial	Reduced complement function	IMD	Susceptibility	Parham et al. (2007)
	c.1599T > A, p.Y493X IVS3 + 3A > C	AR	Familial	Reduced complement function	IMD	Susceptibility	Parham et al. (2007)
	c.1936delG	AR	Familial	Reduced complement function	IMD	Susceptibility	Nishizaka et al. (1996a)
	c.879delG	AR	Familial	Reduced complement function	IMD	Susceptibility	Hobart et al. (1998)
	c.1195delC	AR	Familial	Reduced complement function	IMD	Susceptibility	Zhu et al. (1998)
* C7*	c.2107C > T, p.Q681X	AR	Familial	Reduced complement function	IMD	Susceptibility	Barroso et al. (2010)
	c.2184T > A, p.C728X	AR	Familial	Reduced complement function	IMD	Susceptibility	Nishizaka et al. (1996b)
	c.281-1G > T c.1-?-2350 + ?del	AR	Familial	Reduced complement function	IMD	Susceptibility	Ki et al. (2005)
	c.1135G > C, p. G379R	AR	Familial	Reduced complement function	IMD	Susceptibility	Fernie et al. (1997)
	c.1922delAG	AR	Familial	Reduced complement function	IMD	Susceptibility	Barroso et al. (2004)
	c.633_643del c.1922delAG^a^	AR	Familial	Reduced complement function	IMD	Susceptibility	Barroso et al. (2006)
* C8B*	c.1282C > T, p.R428X	AR	Familial	Reduced complement function	IMD	Susceptibility	Dellepiane et al. (2016)
	c.271C > T, p.Q91X; c.820C > T, p.R274X	AR	Familial	Reduced complement function	IMD	Susceptibility	Saucedo et al. (1995)
	c.1041_1047dup, p.L350fs c.271C > T, p.Q91X^a^	AR	Familial	Reduced complement function	IMD	Susceptibility	Arnold et al. (2009)
* C9*	c.346C > T, p.R116X	AR	Cohort	Reduced complement function	MM	Susceptibility	Kira et al. (1998)
	c.162C > A, p.C54X	AR	Familial	Reduced complement function	IMD	Susceptibility	Zoppi et al. (1990)
* CFD*	c.638T > G, p.V213G; c.640T > C, p.C214R	AR	Familial	Reduced complement function	MS	Susceptibility	Sprong et al. (2006)
	c.125C > A, p.S42X	AR	Familial	Reduced complement function	IMD	Susceptibility	Biesma et al. (2001)
	c.620G > C, p.R176P	AR	Familial	Reduced complement function	IMD	Susceptibility	Sng et al. (2018)
	c. .677–678delinsTTCT c.653T > C, p.L218P^a^	AR	Familial	Reduced complement function	IMD	Susceptibility	El Sissy et al. (2019)
* CFB*	c.766C > T, p.Q256X c.1894-1897delTTTG, p.F632C**fs**X8^a^	AR	Familial	Reduced complement function	MM	Susceptibility	Slade et al. (2013)
* CFI*	c.1282A > T p.H400L	AR	Familial	Reduced complement function	IMD	Susceptibility	Vyse et al. (1996)
	c.1282A > T p.H400L c.801G > A p.del-exon 5^a^	AR	Familial	Reduced complement function	IMD	Susceptibility	Vyse et al. (1996)
	c.266_?_536 + ?del c.1420C > T, p.R474X	AR	Familial	Reduced complement function	IMD	Susceptibility	Alba-Dominguez et al. (2012)
	c.485G > A, p.G162D c.1176_1177dupAT, p.W393Yfs*5	AR	Familial	Reduced complement function	IMD	Susceptibility	Alba-Dominguez et al. (2012)
	c.772G > A, p.A258T	AR	Familial	Reduced complement function	IMD	Susceptibility	Alba-Dominguez et al. (2012)
* C3*	c.1716G > A, p.K552X	AR	Familial	Reduced complement function	IMD	Susceptibility	Da Silva Reis et al. (2002)
* C2*	c.841_868del, p.Val281fs	AR	Cohort	Reduced complement function	IMD	Susceptibility	Jonsson et al. (2005)
**INFLAMMATORY RESPONSE**							
* IRAK4*	c.877C > T, p.Q293X	AR	Case study	Impaired IL-6 production	IMD	Susceptibility	Frans et al. (2015)
* IKBKG*	c.1249T > C, p.C417R	XR	Case study	N/A	IMD	Susceptibility	Huppmann et al. (2015)
* GATA2*	c.988C > T, p.R330X	N/A	Cohort	N/A	IMD	N/A	Spinner et al. (2014)

*IMD* invasive meningococcal disease, *MS* meningococcal septicaemia, *MM* meningococcal meningitis, *AD* autosomal dominant, *XR* X-linked recessive, *AR* autosomal recessive

^a^Compound heterozygous mutations

Surfactant proteins are part of the collectins protein family involved in the innate immune system and in pathogen pattern recognition. They are expressed in the nasopharynx and respiratory tract and can activate inflammatory and phagocyte responses after binding to structures on the microbial cell wall (Pikaar et al. 1995). Surfactant proteins A1 and A2 (SP-A1 and SP-A2, respectively), encoded by *SFTPA1* and *SFTPA2*, respectively, are expressed at the site of *Nm* colonisation. One candidate gene study has exhaustively investigated SP-A proteins in association with IMD describing various polymorphisms both increasing the risk of IMD and also showing a protective effect; however, these findings require further validation by other independent studies (Jack et al. 2006).

## Invasion

Invasion of the nasopharyngeal epithelium leads to dissemination of the bacterium in the bloodstream. The mechanisms that lead to invasion are poorly understood; however, Goldschneider and colleagues in the late 1960s suggested that complement-dependent killing by antibody was a key defence against meningococcal infection, with high antibody titres seen later in life (Goldschneider et al. 1969a). The majority of the population does not develop severe disease, even in those who lack pre-existing bactericidal antibodies, suggesting that the innate immune response plays a key role in preventing invasive disease after meningococcal colonisation of the nasopharynx (Welsch and Granoff 2007). Defects in genes involved in this stage of invasion can provide gaps in host defence and give rise to IMD.

### Complement

**Fig. 2:**
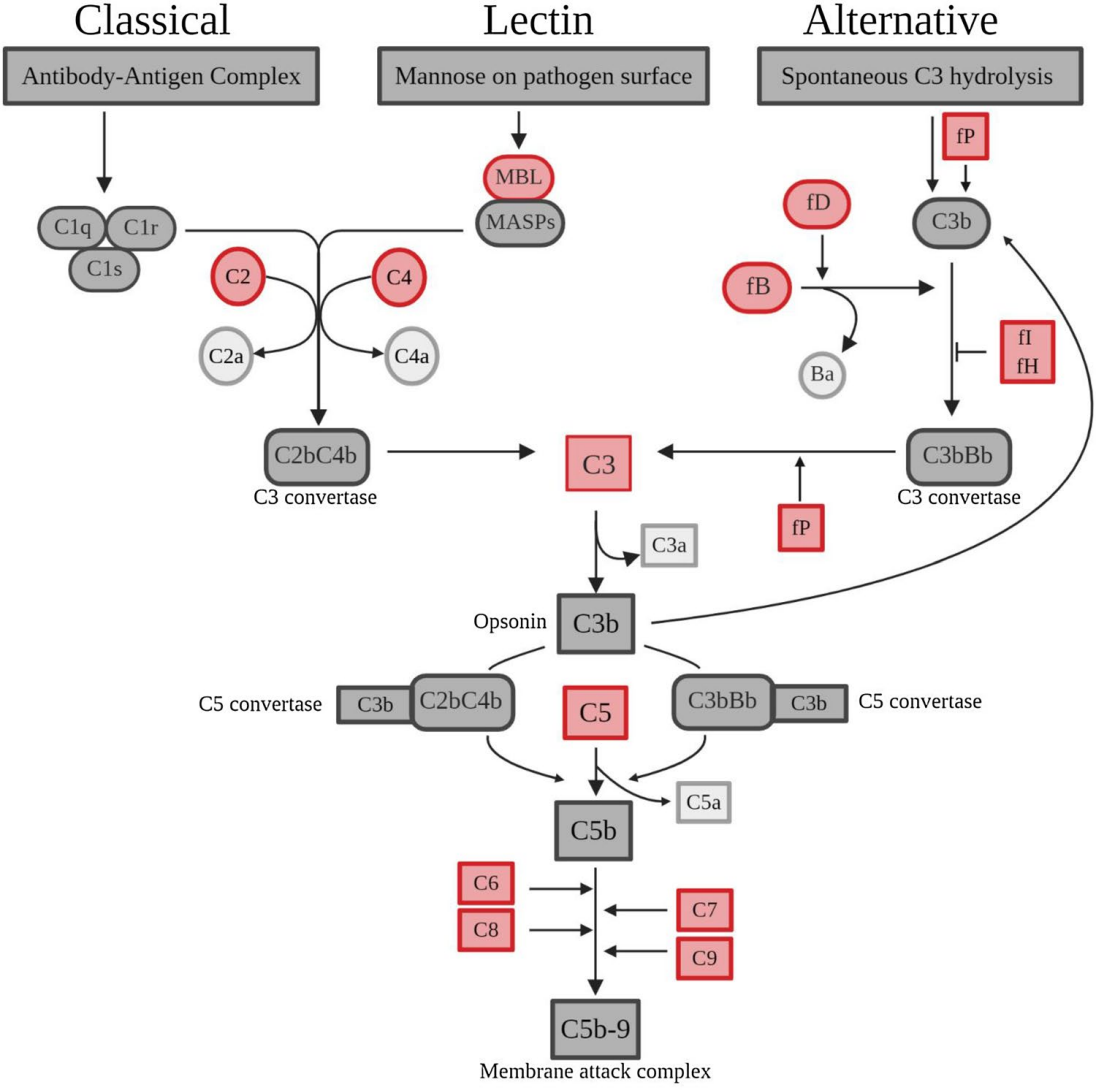
Complement pathway. Overview of three main complement pathways that involves multiple cleavage events that converge to a cleavage of central component C3–C3b, which triggers a cascade that leads to the formation of the membrane-attack complex capable of cell lysis via pore formation. C3b is also an opsonin capable of tagging pathogens for phagocytosis and C3b formation can act as a positive feedback loop for the alternative pathway, necessitating the need for several negative regulators including CFI, and CFH. Properdin is a positive regulator of the alternative pathway, stabilising the C3 convertase. Those in red symbolises factors that have reported loss of function mutations that are associated with either chronic meningococcaemia or IMD. Image created with biorender.com

Complement plays an important role in the innate immune response, assisting in a rapid response against invading pathogens (Lewis and Ram 2014). Complement is activated via three main pathways which all involve complement component 3 (C3): the classical antibody–antigen interaction, the mannose-binding lectin (MBL) interaction with the microbial cell walls and finally, the alternative pathway activated by C3 interacting with complement Factor B (CFB) and complement Factor D (CFD) (Fig. 2) (Janeway et al. 2001). The alternative pathway can also act as an amplification loop for the other two pathways (Janeway et al. 2001). All three pathways feed into the same final pathway of the formation of C3 convertase enzyme that can produce complement component C3b which can act as an opsonin and facilitate phagocytosis by binding to the bacteria. C3b and C3 can also bind to form C5, generating C5b which leads to the formation of the membrane-attack complex (MAC), comprised of complement components C5b–C9, creating pores in the membrane of the bacteria thereby causing bacterial death (Fig. 2) (Heesterbeek et al. 2019; Janeway et al. 2001). Host complement-dependent bactericidal activity is one of the key protective immune responses against meningococcal infection and its role was established early at the start of the twentieth century (Flexner 1913; Flexner and Jobling 1908). Later, Goldschneider and colleagues were able to decisively elucidate the protective role of complement and antibodies against invasive *Nm* infection (Goldschneider et al. 1969a, b). The role of complement as a vital part of host defence against *Nm* infection has been unequivocally established and further supported by the increased susceptibility to infection by complement-deficient individuals described further below (Figueroa et al. 1993).

#### Terminal complement deficiencies

Functional deficiencies of the terminal complement (C5–C9) were one of the first characterised defects associated with IMD in the 1970s and 1980s, whereas the identification of mutations underlying these deficiencies came about later (Lim et al. 1976; Nagata et al. 1989; Petersen et al. 1976; Snyderman et al. 1979). Complement deficiencies can be acquired or inherited, the latter being rarer occurring in 0.03% of the general population with frequencies depending on complement component and ethnicity (Lewis and Ram 2014). Mutations in any one of the terminal complement genes (*C5, C6, C7, C8A, C8B*, or *C9*) result in an autosomal recessive monogenic disorder leading to impaired function of the complement system and increased susceptibility to *Nm* infection (Table 1) (Arnold et al. 2009; Barraso et al. 2004, 2006, 2010; Delgado-Cervino et al. 2005; Dellepiane et al. 2016; Fernie et al. 1997; Figueroa et al. 1993; Hobart et al. 1998; Kaufmann et al. 1993; Ki et al. 2005; Kira et al. 1998; Kojima et al. 1998; Lee et al. 1978; Lewis and Ram 2014; Marujo et al. 2019; Nishizaka et al. 1996a, b; Owen et al. 2015; Parham et al. 2007; Pfarr et al. 2005; Platonov et al. 1993; Saucedo et al. 1995; Wang et al. 1995; Wurzner et al. 1995; Zhu et al. 1998; Zoppi et al. 1990). Patients with deficiencies of the terminal complement are characteristically distinct as they typically present with recurrent meningococcal infection, with lower mortality rates per episode (Figueroa and Densen 1991; Fijen et al. 1999; Platonov et al. 1993).

#### Alternative pathway complement factors

Functional deficiencies of all the alternate pathway factors have been associated with IMD. Properdin is a positive regulator of the alternative complement pathway by binding to and stabilising C3b, prolonging its half-life and functional activity, as well as functioning as an initiator of the alternative pathway (Lewis and Ram 2014). Functional properdin deficiency resulting in impaired complement response and reduced bactericidal activity was first associated with IMD in a multiplex kindred in the 1980s (Braconier et al. 1983; Cunliffe et al. 1995; Densen et al. 1987; Figueroa and Densen 1991; Fijen et al. 1989; Genel et al. 2006; Nielsen and Koch 1987; Nielsen et al. 1989; Ross and Densen 1984; Schlesinger et al. 1990, 1993; Sjoholm et al. 1982; Spath et al. 1999). Genetic deficiency of properdin, encoded by *CFP*, is an X-linked recessive disorder and is typically associated with non-recurrent and rapidly progressive fatal meningococcaemia (Table 1) (Fredrikson et al. 1996; Helminen et al. 2012; Sjoholm et al. 1982; Spath et al. 1999; van den Bogaard et al. 2000; Westberg et al. 1995). In properdin-deficient patients, around 50% of IMD is caused by uncommon serogroups of *Nm* such as W and Y (Figueroa and Densen 1991)*.* Functional deficiency of complement factor D (CFD) was first reported in a patient with recurrent *Nm* infections (Hiemstra et al. 1989). The discovery of mutations in *CFD* resulting in an autosomal recessive disorder predisposing to IMD was subsequently reported in other unrelated kindreds (Table 1) (Biesma et al. 2001; El Sissy et al. 2019; Sng et al. 2018; Sprong et al. 2006). There has been one report of autosomal recessive complement Factor B (CFB) deficiency with recurrent pneumococcal and meningococcal infections (Table 1) (Slade et al. 2013). Complement Factor I (CFI) is a negative regulator of the alternative pathway that proteolytically inactivates C3b. CFI deficiency results in uncontrolled continuous activation of the alternative pathway and is associated with recurrent infections from encapsulated bacteria (Alba-Dominguez et al. 2012). In the 1990s, a study suggested CFI deficiency, resulting from recessive mutations, to be responsible for two cases of recurrent pyogenic infections, including *Nm* infection (Vyse et al. 1996). More recently several patients with autosomal recessive CFI deficiency have been associated with IMD (Table 1) (Alba-Dominguez et al. 2012).

Finally, GWASs have identified polymorphisms in a broad region spanning complement factor H (*CFH*) and complement factor H-related 3 (*CFHR3)* as highly significantly associated with IMD (Table 2) (Davila et al. 2010; Martinon-Torres et al. 2016). This association has been validated in different cohorts and represents the most significant genetic association with susceptibility to IMD (Table 2) (Bradley et al. 2015; Davila et al. 2010; Haralambous et al. 2006; Martinon-Torres et al. 2016). CFH acts as a negative regulator and competes with CFB resulting in inactive C3b (Fig. 2) (Janeway et al. 2001).

**Table 2:** Polymorphisms associated with meningococcal disease

Gene	Variant	Genetic model	Significance	Study type	Gene-specific phenotype	Infection phenotype	Disease outcome	References
**COLONISATION**								
* CEACAM3*	Haplotype C^d^	Additive	*P* < 0.001 OR = 0.52 (95% CI 0.35–0.075)	Candidate gene	N/A	IMD	Protective	Callaghan et al. (2008)
* CEACAM6*	Haplotype B^d^	Additive	*P* < 0.001 OR = 0.29 (95% CI 0.14–0.61)	Candidate gene	N/A	IMD	Protective	Callaghan et al. (2008)
	Haplotype C^d^	Additive	*P* = 0.018 OR = 2.01 (95% CI 1.13–3.6)	Candidate gene	N/A	IMD	Susceptibility	Callaghan et al. (2008)
* SFTPA2*	rs1059046 (REF); rs17886395 (REF);rs1965707; rs1965708	Recessive	*P* = 0.025 OR = 7.4 (95% CI 1.3–42.4)	Candidate gene	N/A	IMD	Susceptibility	Jack et al. (2006)
	rs1059046 (REF); rs17886395; rs1965707; rs1965708 (REF)	Dominant	*P* = 0.045 OR = 0.3 (95% CI 0.1–0.97)	Candidate gene	N/A	IMD	Protective	Jack et al. (2006)
	rs1965708	Recessive	*P* = 0.016 OR = 6.7 (95% CI 1.4–31.5)	Candidate gene	N/A	IMD	Susceptibility	Jack et al. (2006)
	rs1965708	Recessive	OR = 2.9 (95% CI 1.1–7.7)	Candidate gene	N/A	Death	Susceptibility	Jack et al. (2006)
**INVASION**								
* CFHR3*	rs426736	Additive	*P* = 4.6 × 10^–13^ OR = 0.63 (95% CI 0.55–0.71)	GWAS	N/A	IMD	Susceptibility	Davila et al. (2010)
* CFH*	c.-496C > T (REF)	Recessive	*P* = 0.001 OR = 2.0 (95% CI 1.3–3.2)	Candidate gene	High fH levels and reduced bactericidal activity	IMD	Susceptibility	Haralambous et al. (2006)
	rs1065489	Additive	*P* = 2.2 × 10^–11^ OR = 0.64 (95% CI 0.56–0.73)	GWAS	N/A	IMD	Susceptibility	Davila et al. (2010)
	rs1061170	Dominant	*P* = 5.3 × 10^–3^ OR = 1.26 (95% CI 1.07–1.49)	Candidate gene	N/A	IMD	Susceptibility	Bradley et al. (2015)
	rs3753396	Dominant	*P* = 3.0 × 10^–5^ OR = 0.56 (95% CI 0.43–0.74)	Candidate gene	N/A	IMD	Protective	Bradley et al. (2015)
* MBL2*	rs5030737; rs1800450; rs1800451	Dominant	*P* < 0.001	Candidate gene	N/A	IMD	Susceptibility	Faber et al. (2007)
	rs5030737; rs1800450; rs1800451	Dominant	*P* = 0.001 OR = 2.0 (95% CI 1.3–3.0)	Candidate gene (hospital cohort)	N/A	IMD	Susceptibility	Hibberd et al. (1999)
	rs5030737; rs1800450; rs1800451	Dominant	*P* = 0.008 OR = 2.4 (95% CI 1.2–4.6)	Candidate gene (community-based study)	N/A	IMD	Susceptibility	Hibberd et al. (1999)
**INFLAMMATORY RESPONSE**								
* TLR4*	rs4986790	Dominant	*P* = 0.021 OR = 3.3 (95% CI 1.14–9.73)	Candidate gene	N/A	Death	Susceptibility	Faber et al. (2009)
	rs4986790	Dominant	*P* = 0.006 OR = 3.003 (95% CI 1.331–6.775)	Candidate gene	N/A	IMD	Susceptibility	Faber et al. (2006)
* TLR9*	rs352140	Dominant	*P* = 0.0098 OR = 0.6 (95% CI 0.4–0.9)	Candidate gene	N/A	MM	Protective	Sanders et al. (2011)
* TNF*	rs1800629	Dominant	*P* = 0.03 RR = 2.5 (95% CI 1.1–5.7)	Candidate gene	High TNF-α	Death	Susceptibility	Nadel et al. (1996)
	rs1800629	Dominant	*P* = 0.02 RR = 1.6 (95% CI 1.1–2.3)	Candidate gene	High TNF-α	IMD	Susceptibility	Nadel et al. (1996)
	rs1800629 (REF)	Dominant	OR = 3.619 (95% CI 1.758–7.449)	Candidate gene	N/A	IMD	Susceptibility	Titmarsh et al. (2013)
	rs1800629 (REF)	Recessive	OR = 3.791 (95% CI 1.720–8.357)	Candidate gene	N/A	IMD	Susceptibility	Titmarsh et al. (2013)
	rs1800629	Recessive	OR = 1.93 (95% CI 1.08–3.46)	Candidate gene	High TNF-α	IMD	Susceptibility	Read et al. (2009)
* IL1B*	rs16944 (REF)	Recessive	*P* < 0.001 OR = 3.39 (95% CI 1.39–8.29)	Candidate gene	N/A	Death	Increased severity	Read et al. (2000)
	rs16944	Recessive	*P* < 0.001 OR = 7.35 (95% CI 2.51–21.45)	Candidate gene	N/A	Death	Increased severity	Read et al. (2000)
	rs16944 (REF)	Dominant	*P* = 0.023 OR = 2.05 (95% CI 1.10–3.79)	Candidate gene	N/A	Death	Protective	Read et al. (2003)
* IL1B/IL1RN*	rs16944/rs419598 (REF)	Dominant/Recessive	*P* = 0.018 OR = 7.78 (95% CI 1.05–59.05)	Candidate gene	N/A	Death	Protective	Read et al. (2000)
* IL1B/IL1RN*	rs16944/rs419598	Dominant/Dominant	OR = 0.61 (95% CI 0.38–0.993)	Candidate gene	N/A	Death	Susceptibility	Read et al. (2003)
* IL1RN*	86-basepair VNTR	Recessive	*P* = 0.033–0.043	Candidate gene	N/A	IMD	Susceptibility	Balding et al. (2003)
	rs419598	Recessive	OR = 2.0 (95% CI 1.1–3.4)	Candidate gene	N/A	IMD	Susceptibility	Endler et al. (2006)
* IL6*	rs1800795 (REF)	Recessive	OR = 2.64 (95% CI 1.12–6.22)	Candidate gene	N/A	Death	Susceptibility	Balding et al. (2003)
	rs1800795 (REF)	Recessive	OR = 4.395 (95% CI 1.900–10.162)	Candidate gene	N/A	IMD	Susceptibility	Titmarsh et al. (2013)
* IL10*	rs1800896	Recessive	*P* = 0.0078 OR = 2.7 (95% CI 2.3–3.6)	Candidate gene	N/A	IMD	Susceptibility	Balding et al. (2003)
**ACQUIRED IMMUNITY**								
* FCGR2A*	rs1801274	Recessive	*P* = 0.028 OR = 2.67 (95% CI 1.09–6.53)	Candidate gene	Reduced phagocytosis	MS	Susceptibility	Bredius et al. (1994)
	rs1801274	Recessive	*P* < 0.03 OR = 2.9 (95% CI 1.1–7.3)	Candidate gene	N/A	IMD	Susceptibility	Platonov et al. (1997)
	rs1801274 (REF)	Recessive	*P* < 0.02 OR = 4.7 (95% CI 1.5–14.5)	Candidate gene	N/A	IMD	Protective	Platonov et al. (1997)
	rs1801274	Dominant	*P* < 0.01 OR = 14	Candidate gene	N/A	IMD	Susceptibility	Platonov et al. (1998)
	rs1801274	Recessive	*P* = 0.04 OR = 3.9 (95% CI1.0–16)	Candidate gene	N/A	IMD	Susceptibility	Domingo et al. (2002)
	rs1801274	Recessive	*P* = 0.004 OR = 3 (95% CI 1.4–7.8)	Candidate gene	N/A	IMD	Susceptibility	Domingo et al. (2002)
	rs1801274	Recessive	*P* = 0.03 OR = 3.5 (95% CI 1.1–11.7)	Candidate gene	N/A	IMD	Susceptibility	Domingo et al. (2004)
* FCGR2A/FCGR3B*	rs1801274/NA2 allotype	Recessive	*P* = 0.036 OR = 13.9 (95% CI 1.2–478)	Candidate gene	Reduced phagocytosis	IMD	Susceptibility	Fijen et al. (2000)
**COAGULATION AND FIBRINOLYSIS**								
* F5*	rs6025	Dominant	*P* < 0.03 RR = 3.1 (95% CI 1.2–7.9)	Candidate gene	Increased thrombosis	Purpura	Increased severity	Kondaveeti et al. (1999)
						fulminans		
	rs6025	Recessive	Single case study	Candidate gene	Increased thrombosis	Purpura	Increased severity	Sackesen et al. (1998)
						fulminans		
* SERPINE1*	rs1799889	Recessive	RR = 2.0 (95% CI 1.0–3.8)	Candidate gene	Higher PAI-1 concentration	Death	Increased severity^a^	Hermans et al. (1999)
	rs1799889	Recessive	*P* = 0.005 RR = 1.9 (95% CI 1.2–3.0)	Candidate gene	N/A	Death	Increased severity^a^	Haralambous et al. (2003)
	rs1799889	Recessive	*P* < 0.0001 RR = 2.7 (95% CI 1.6–4.6)	Candidate gene	N/A	MS	Increased severity^a^	Haralambous et al. (2003)
	rs1799889	Recessive	*P* = 0.03 RR = 2.4 (95% CI 1.1–5.0)	Candidate gene	N/A	Vascular complications	Increased severity^a^	Haralambous et al. (2003)
	rs1799889	Recessive	OR = 5.9 (95% CI 1.9–18.0)	Candidate gene	N/A	MS	Increased severity^a^	Westendorp et al. (1999)
	rs1799889	Recessive	*P* = 0.001	Candidate gene	N/A	MM	Increased severity^b^	Westendorp et al. (1999)
	rs1799889	Recessive	*P* = 0.037 OR = 2.31 (95% CI 1.04–5.14)	Candidate gene	N/A	Death	Increased severity^a^	Geishofer et al. (2005)
	rs1799889	Recessive	*P* = 0.01 OR = 2.21 (95% CI 1.20–4.08)	Candidate gene	N/A	MS	Increased severity^a^	Geishofer et al. (2005)
	rs1799889	Recessive	*P* = 0.014 HR 1.5 (95% CI 1.1–2.1)	Candidate gene	N/A	Disseminated intravascular coagulation	Increased severity^a^	Binder et al. (2007a)
* PROC*	rs1799808 (REF), rs1799809	Recessive	*P* = 0.04	Candidate gene	Low protein C levels ^c^	IMD	Susceptibility	Binder et al. (2007b)
	rs1799808, rs1799809	Dominant	*P* = 0.036 OR = 3.43 (95% CI 1.05–11.20)	Candidate gene	N/A	MS	Increased severity	Binder et al. (2007b)
	rs1799808, rs1799809 (REF)	Recessive	*P* = 0.017 OR = 0.09 (95% CI 0.01–0.94)	Candidate gene	N/A	MS	Protective	Binder et al. (2007b)
* CPB2*	rs779491029	Recessive	*P* = 0.03 OR = 3.1 (95% CI 1.0–9.5)	Candidate gene	N/A	Death	Increased severity	Kremer Hovinga et al. (2004)
	rs779491029	Recessive	OR = 13.7 (95% CI 1.5–123)	Candidate gene	Increased anti-fibrinolytic activity	MS	Increased severity	Emonts et al. (2008)

All alleles refer to alternative alleles unless denoted (REF)for referent allele

*IMD* meningococcal disease, *MS* meningococcal septicaemia, *MM* meningococcal meningitis, *OR* odds ratio, *RR* risk ratio, *HR* hazard ratio.

^a^4G/4G genotype

^b^5G/5G genotype

^c^Function of variant reported (Brandtzaeg et al. 1989)

^d^Refer to reference for individual SNPs

The mechanisms underlying the association of polymorphisms in the *CFH/CFHR3* region with IMD have begun to be clarified. *Nm* expresses a factor H-binding protein (fHBP) on its surface which binds human CFH. This binding assists the bacteria in evading complement-mediated killing in the blood stream (Schneider et al. 2009). CFHR proteins, which have partial homology to CFH, can antagonise the immune evasion through competition for fHBP binding (Caesar et al. 2014). However, the plasma concentrations of the CFHR proteins are low compared to that of CFH (Pouw et al. 2016), and patients with deletions in the *CFHR* region are not at increased risk of IMD (Davila et al. 2010). Therefore, other mechanisms are likely to contribute to the association of the *CFH/CFHR3* region with IMD (Caesar et al. 2014). The EUCLIDS consortium (Martinon-Torres et al. 2018) is currently exploring the role of genetic polymorphisms in the *CFH/CFHR3* region in determining CFH plasma concentrations.

#### Early complement components

Deficiencies of the early complement components, C1, C2, and C4, are inherited in an autosomal recessive manner and classically associated with autoimmune diseases, particularly systemic lupus erythematosus (SLE), although the impact on *Nm* infection is controversial (Fijen et al. 1989; Macedo and Isaac 2016; Tebruegge and Curtis 2008). The role of the C4 isoforms (C4A and C4B) in *Nm* infection is conflicting with reports that C4 deficiency alone is not significant enough to predispose individuals to bacterial infection (Bishof et al. 1990; Cates et al. 1992; Fasano et al. 1990). Until 1991, only six cases of C2 deficiency with incidence of IMD were reported (Figueroa and Densen 1991). Since then, few cases of IMD patients with C2 deficiency have been reported including a 4-year-old child from England, a 12-year-old child suffering from primary meningococcal arthritis as a result of *Nm* serogroup Y infection, and three patients from Sweden with homozygous C2 deficiency (Hoare et al. 2002; Hussain et al. 2007; Jonsson et al. 2005). Primary C3 deficiencies are rare, most likely due to the central role, it plays in the complement response, but these patients can suffer from recurrent bacterial infections, including from *Nm* (Da Silva Reis et al. 2002).

#### Mannose-binding lectin

MBL is a collectin, encoded by *MBL2*, that can recognise *Nm* and trigger the complement cascade by forming a complex and binding mannose residues present on pathogen surfaces (Janeway et al. 2001). In a candidate gene study, three functional variants in codon 52, 54, and 56 of *MBL2* exon 1 show reduced plasma protein concentrations and have been previously associated with IMD (Table 2) (Hibberd et al. 1999). *MBL2* polymorphisms were also found to be significantly associated with IMD in a paediatric cohort with IMD incidence increasing with younger age (Faber et al. 2007); however, in a subsequent study, these polymorphisms were found to have no significant association with IMD (Lundbo et al. 2015). No association was also found between low serum MBL concentrations and serogroup B/C IMD in a Norwegian cohort (Garred et al. 1993).

## Inflammatory response

The proper induction of the immune response including activation of immune cells and cytokines following infection is critical for preventing IMD as is demonstrated by the description of IMD in a patient with a mutation in *GATA2*, a hematopoietic transcription factor, resulting in cytopenias and associated with viral and bacterial infections and malignancies (Table 1) (Spinner et al. 2014). Cytokine production is regulated by a complex system involving multiple factors and mediators (Westendorp et al. 1997). Some mutations can dysregulate this process and the immunological phenotype can vary. *IRAK4* and *NEMO* deficiencies result in reduced cytokine levels including an abolished IL-6 response (Picard et al. 2011; von Bernuth et al. 2008). Conversely, some variants of *IL1B* and *TNF*, can result in an excessive inflammatory response that can increase risk of developing severe disease and even death in meningococcal infection (Nadel et al. 1996; Read et al. 2000, 2003). Key cytokines in IMD include the pro-inflammatory cytokines interleukin-1-beta (IL-1β), interleukin-6 (IL-6), tumour necrosis factor-alpha (TNF-α), and the anti-inflammatory cytokines IL-10 and IL-1 receptor antagonist (IL-1Ra) (Hackett et al. 2001; Pathan et al. 2003). These key cytokines have been investigated in candidate gene studies in relevance to IMD.

### Toll-like receptors

The toll-like receptor (TLR) signalling pathway is a central part of the innate immune response as it recognises pathogens, triggering a signalling cascade that ends in production of cytokines and chemokines (Kawai and Akira 2011). Genetic deficiencies of key mediators of the innate immune response, autosomal recessive *IRAK4*, and X-linked recessive *IKBKG* (encoding for NEMO) deficiencies, underlie pyogenic bacterial infection with impaired interleukin-6 (IL-6) production and C-reactive protein (CRP) production (Ku et al. 2007; Picard et al. 2003, 2011). Deficiencies in these proteins are associated with impaired canonical TLR signalling pathway and typically predispose to pyogenic bacterial infections; however, cases of IMD have also been observed (Frans et al. 2015; Huppmann et al. 2015; Picard et al. 2010, 2011). Other polymorphisms associated with IMD have been identified in specific TLRs, including *TLR4* and *TLR9*. TLR4 is a major transmembrane receptor expressed in immune cells and recognises bacterial lipopolysaccharides (LPS), a major outer membrane component of Gram-negative bacteria including *Nm* (Kawai and Akira 2011). Binding of LPS to the TLR4 complex initiates a signalling cascade leading to the activation of NF-κB-mediated transcription and production of pro-inflammatory cytokines (TNF, IL6, IL1 etc.) (Kawai and Akira 2011). A candidate gene-based study found an excess of rare heterozygous missense mutations in *TLR4* in a cohort of patients with IMD (Smirnova et al. 2003). A *TLR4* polymorphism, (rs4986790), results in hypo-responsiveness to LPS (Arbour et al. 2000) which has been associated with IMD, and mortality (Table 2) (Faber et al. 2006, 2009), with conflicting findings (Biebl et al. 2009; Read et al. 2001). A candidate gene study-associated polymorphisms in *TLR9*, an intracellular, endosomal, receptor that recognises unmethylated CpG motifs in bacterial DNA, with meningococcal meningitis in a large paediatric cohort (Table 2) (Kawai and Akira 2011; Sanders et al. 2011).

### Cytokine response

TNF-α is central to the activation of the inflammatory response, it mediates septicaemia and septic shock and circulating TNF-α is correlated to severity, and mortality, in IMD (Waage et al. 1987). Possession of the rare *TNF* allele, resulting from a single nucleotide polymorphism (SNP) in the promoter region (rs1800629), was shown to increase constitutive and inducible secreted TNF-α and may predispose to susceptibility and severity to *Nm* infection (Table 2) (Nadel et al. 1996; Read et al. 2009). However, another study has reported that it is the referent GG genotype that increases risk of IMD (Table 2) (Titmarsh et al. 2013) whereas other studies report no association between *TNF* and IMD (Balding et al. 2003; Domingo et al. 2004; Read et al. 2000), showing that these results have not been reproducible and more work is needed to determine its effect. IL-6 is secreted by macrophages and T cells and has pro-coagulant effects that assist in the regulation of the immune response and haematopoiesis (Tanaka et al. 2014). High levels of circulating IL-6 are associated with poor outcome in meningococcal septic shock and septicaemia (Hack et al. 1989; Waage et al. 1989). A particular SNP in *IL-6* (rs1800795) has been associated with an increased risk of death in IMD (Table 2) (Balding et al. 2003; Titmarsh et al. 2013). IL-10 is an anti-inflammatory cytokine that suppresses the inflammatory response, upregulates IL-1Ra, and downregulates other pro-inflammatory cytokines. A SNP in *IL-10* (rs1800896) has been significantly correlated with severe disease in IMD (Table 2) (Balding et al. 2003).

IL-1α and IL-1β are pro-inflammatory cytokines, produced mainly by macrophages and monocytes that binds to the IL-1 receptor (IL-1R) complex and activates the acute phase response. IL-1Ra, encoded by *IL-1RN*, can also compete with the binding of IL-1α and IL-1β to the IL-1R complex. Increased levels of IL-1β and IL-1Ra have been associated with IMD. Allelic variation at the IL-1 gene cluster affects the inflammatory response and course of infection (Read et al. 2003). A SNP in *IL1B* (rs16944) has been associated with an increased risk of death in homozygous individuals (Table 2) (Read et al. 2000, 2003). Furthermore, the presence of the heterozygous *IL1B* (rs16944) polymorphism combined with the homozygous *IL-1RN* (rs419598) polymorphism is also associated with outcome of IMD (Table 2) (Read et al. 2000, 2003). However, another study described no association between the *IL1B* (rs16944) polymorphism and IMD outcome but showed that IMD outcome was associated with the *IL1RN* homozygous (rs419598) polymorphism (Table 2) (Endler et al. 2006). An 86 base pair variable number tandem repeat (VNTR) in intron 2 of *IL1RN* has also been associated with mortality and severe septicaemia in IMD patients (Table 2) (Balding et al. 2003).

## Acquired immunity

Fc receptors for IgG (FcγR) are found on phagocytes and are a central component for phagocytosis. Three subclasses of FcγRs exhibit biallelic polymorphisms that influence the IgG-binding efficiency. FcγRIIa, FcγRIIIa, and FcγRIIIb, encoded by *FCGR2A*, *FCGR3A*, and *FCGR3B*, respectively, are shown to be important against meningococcal infection (Fijen et al. 2000; van der Pol et al. 2001). FcγRIIa is expressed on poly-morphonuclear cells and is the only subclass that can bind IgG2 (van der Pol et al. 2001). There are two FcγRIIa allotypes determined by rs1801274 (p.H131R), in humans the allotype FcγRIIa-H/H131 is more effective at IgG2-mediated phagocytosis of encapsulated bacteria (Sanders et al. 1995). Multiple candidate gene studies have shown correlation of the *FCGR2A* rs1801274 (p.H131R) polymorphism in development of severe IMD in patients (Table 2) (Bredius et al. 1994; Domingo et al. 2002, 2004; Platonov et al. 1997, 1998). Patients with the FcγRIIa-H/H131 allotype have been reported to have higher antibody-mediated phagocytosis-dependent resistance to IMD compared to patients carrying the FcγRIIa-R/R131 allotype and were less likely to develop severe IMD (Platonov et al. 1997, 1998). There have also been conflicting studies showing no association between FcγRIIa p.H131R and IMD (Smith et al. 2003; van der Pol et al. 2001). FcγRIIIa is expressed on monocytes, macrophages and natural killer cells, and can bind IgG1, IgG3, and IgG4. There are two allotypes determined by *FCGR3A* rs396991 (p.V158F), with the V158 allotype able to increase binding of IgG (Koene et al. 1997). Finally, FcγRIIIb is expressed on neutrophils and binds IgG1, and IgG3. FcγRIIIb contains a neutrophil antigen polymorphism (NA1/NA2), attributed to a group of five base substitutions, with FcγRIIIb-NA1 shown to bind more efficiently than FcγRIIIa-NA2 (van der Pol et al. 2001). FcγRIIIb polymorphism alone is not associated with IMD but a combination of homozygous polymorphisms of all three FcγRs is observed in candidate gene studies to be significantly increased in relatives of IMD patients (Smith et al. 2003; van der Pol et al. 2001). Furthermore, a homozygous combination of *FCGR2A* p.H131R and *FCGR3B* NA2 was reported in a Dutch cohort of terminal complement deficient families to be associated with IMD (Table 2) (Fijen et al. 2000).

## Coagulation and fibrinolysis

Circulating meningococcal endotoxin is a strong activator of the coagulation pathway causing generation of thrombin (Lecuyer et al. 2017). Coagulopathy is a feature of severe IMD, resulting in meningococcal shock which can lead to the most severe complication, purpura fulminans, developing in 15–20% of cases (Kondaveeti et al. 1999; Powars et al. 1993). Purpura fulminans is primarily a thrombotic disorder that is characterised by widespread intravascular thrombosis and haemorrhagic lesions that can progress into skin necrosis requiring grafting or amputations (Kondaveeti et al. 1999; Lecuyer et al. 2017; Powars et al. 1993). The fibrinolytic system can regulate the coagulation response but polymorphisms in genes that are part of coagulation and fibrinolysis can deregulate this interaction and result in IMD (Lecuyer et al. 2017). Most candidate gene studies of genes involved in this pathway investigate the severity of IMD by comparing more severe manifestations of IMD, such as death or purpura fulminans, against non-severe IMD. The factor V Leiden mutation (FV^L^), a SNP in *F5* rs6025, is associated with thrombotic events (Kondaveeti et al. 1999). This polymorphism also results in resistance to activated protein C, a key anti-coagulant that can inhibit plasminogen activator inhibitor (PAI) and deactivate factor V, and factor VIII, to downregulate a pro-coagulation signalling cascade. The FV^L^ mutation has been associated with development of severe purpura fulminans in IMD as a homozygous mutation in a single case study and as a heterozygous mutation in a large paediatric cohort candidate gene study (Table 2) (Kondaveeti et al. 1999; Sackesen et al. 1998). Protein C, encoded by *PROC* gene, is activated by thrombin and in meningococcal septicaemia low protein C plasma levels are associated with increased disease severity (Brandtzaeg et al. 1989). Two SNPs, in the *PROC* 5′UTR promoter region (rs1799808 and rs1799809), are known to affect activated protein C plasma levels (Spek et al. 1995). In a candidate gene study, a specific *PROC* haplotype was associated with IMD (Table 2) (Binder et al. 2007b). Furthermore, the authors suggested that possession of the CG haplotype increased the risk of developing meningococcal septicaemia and that a homozygous TA haplotype conferred protection against meningococcal septicaemia (Table 2) (Binder et al. 2007b).

A polymorphism in thrombin activatable fibrinolysis inhibitor (TAFI), encoded by *CPB2* gene, rs779491029, has been shown to increase its anti-fibrinolytic potential (Emonts et al. 2008). This candidate gene study demonstrated an association of the polymorphism with an increased risk of developing septicaemia and was observed in the parents of IMD fatalities (Table 2) (Emonts et al. 2008; Kremer Hovinga et al. 2004; Schneider et al. 2002). Furthermore, a 4G/5G insertion/deletion polymorphism in the *SERPINE1* promoter region was found to determine plasma PAI-1 levels and promotes severe coagulopathy, with high levels of PAI-1 associated with severe meningococcal septic shock and poor outcome of IMD (Table 2) (Binder et al. 2007a; Geishofer et al. 2005; Hermans et al. 1999; Westendorp et al. 1999). The 4G/4G genotype is associated with increased plasma PAI-1 levels and mortality in severe adult septicaemia (Lorente et al. 2015). A candidate gene study of the relatives of IMD patients reported that the homozygous 4G genotype was associated with meningococcal septic shock, whereas the 5G homozygous genotype was associated with meningococcal meningitis (Table 2) (Westendorp et al. 1999). However, a meta-analysis study has shown no association to be found between the *SERPINE1* promoter polymorphism and sepsis susceptibility (Shi et al. 2015).

## Conclusion

There is strong evidence for a central role for host genetics in predisposition to meningococcal infection. Common polymorphisms, by dint of their frequency, may play a large role when the interaction between pathogen and host is considered at a population level. However, rare monogenic disorders are most significant for an individual, and they provide unprecedented insight into disease mechanisms. To date, mutations in genes involved in complement pathways continues to appear in all host genetic investigations of *Nm* infection, indicating a key role for complement in host defence against infection. Both rare monogenic traits, such as terminal complement deficiencies of *C5-C9, CFD, CFB, CFI,* and *C3*, and common polymorphisms in the *CFH*/*CFHR3* region have been found in association with IMD. The large majority of genes discussed in this review were discovered through candidate gene studies, and most findings require validation in larger studies. Furthermore, caution must be taken interpreting SNP findings that have not been validated in other populations as significant findings resulting from candidate gene studies may in part relate to haplotype variation between populations where SNPs are found. Candidate gene studies are being largely replaced by the more robust GWAS and large-scale sequencing studies. Future genetic studies may focus on meningococcal strain-specificity, elaborate on disease-outcome specific associations, and include a better understanding of the effect size(s) contributed by a single or combination of variants/mutations in IMD which can help in estimating clinical risk of developing IMD at the individual level. In the UK, the recent introduction of the 4CMenB vaccine has reduced but not eliminated IMD (Parikh et al. 2016); however, the efficacy in protecting those with underlying immunodeficiencies remains unknown (Gianchecchi et al. 2015). The most vulnerable patients who develop IMD may contribute to vaccine failures due to the nature of their immunodeficiencies as observed in invasive pneumococcal disease (Maglione et al. 2014). Hence, the significance of understanding the underlying genetics of IMD is as relevant as ever. Given the increasing availability of patient-based genetic sequencing, we propose that children who have had a single severe episode of IMD should be considered for genetic investigations. Currently, the authors are exploring whether detailed genetic investigations on a patient by patient basis is a useful adjunct to the follow-up care of patients with IMD. Identification of key pathways for protection against meningococcal infection will contribute vital knowledge to our understanding of the pathogenesis of IMD.
